# Cocoa Pod Husk Valorization Through *Rhizopus stolonifer* Solid-State Fermentation: Enhancement in Antioxidant Activity

**DOI:** 10.3390/microorganisms13040716

**Published:** 2025-03-22

**Authors:** Patrick Barros Tiburcio, Dão Pedro de Carvalho Neto, Carlos Ricardo Soccol, Adriane Bianchi Pedroni Medeiros

**Affiliations:** 1 Department of Bioprocess Engineering and Biotechnology, Federal University of Paraná (UFPR), Curitiba 82590-300, Brazil; barrostiburcio@ortho.wisc.edu (P.B.T.); soccol@ufpr.br (C.R.S.); 2 Department of Biotechnology, Federal Institute of Paraná (IFPR), Londrina 86077-080, Brazil; daopcn@gmail.com

**Keywords:** cocoa pod husk, solid-state fermentation, sustainable biotechnology, food microbiology

## Abstract

Cocoa pod husk (CPH), a significant agricultural byproduct of cocoa production, presents an opportunity for sustainable valorization through biotechnological methods. This study aimed to enhance the nutritional, antioxidant, and therapeutic properties of CPH using solid-state fermentation (SSF) with *Rhizopus stolonifer*. Physicochemical characterization confirmed CPH’s suitability for SSF, providing a nutrient-rich and favorable environment for fungal growth. The fermentation process significantly improved protein recovery (11.327 ± 0.859 mg g^−1^) and antioxidant activity, with ORAC (51.68 ± 0.35 mmol TE g^−1^) and DPPH (7.09 ± 0.05 µmol TE g^−1^) assays demonstrating marked increases in redox potential, particularly at 144 h and 96 h of fermentation, respectively. GC-MS analysis revealed the generation of bioactive compounds in fermented CPH (CPHF), including methyl 3-hydroxybutyrate, 10,12-Tricosadiynoic acid, and palmitic acid, which are known for their antioxidant, anti-inflammatory, and therapeutic properties. Additionally, phenolic compounds are biotransformed into more bioavailable forms, further enhancing the functional value of the material. This work demonstrates that SSF can effectively transform CPH from an agricultural waste product into a high-value biomaterial with potential applications in functional food, nutraceutical, and pharmaceutical industries. By addressing waste management challenges and promoting the development of innovative bio-based products, this study highlights the promising role of SSF in advancing sustainable and circular biotechnological solutions.

## 1. Introduction

Cocoa is an essential commodity in the world economic scenario, with a global gross production in 2023/2024 year (October–September) of almost 4.5 million tons [1] and an economic driving force for the major producing countries, such as Brazil, where processors handled over 220 thousand tons in 2023 and over 58 thousand tons in the first semester of 2024 [2]. However, the highly valuable cocoa beans used primarily in the chocolate industry constitute only 10% of the cocoa fruit’s dry weight [3]. The remaining lignocellulosic constituents (e.g., cocoa pod husk and bean shell) are systematically generated at a 10:1 ratio and contribute significantly to the approximately 220 billion tons of residual biomass generated annually worldwide [4].

The cocoa pod husk (CPH) is a thick, oval-shaped external layer of the fruit. It is characterized by its constitution rich in non-starch polysaccharides (cellulose, hemicelluloses, and pectin), terpenoids, flavonoids, phenolic, and carboxylic acids (protocatechuic, salicylic, citric, and tartaric acids) [5]. Even though CPH is generally used as a fertilizer due to its high mineral and organic content, the incorrect disposal of this residual biomass presents an environmental concern. CPH may act as a reservoir of phytopathogens, such as *Phytophthora* sp., which are responsible for black pod rot disease [3,6].

In this sense, the biotechnological exploitation of CPH could improve its composition quality, support its valorization, and reduce its environmental impact [6]. The microbiological process of solid-state fermentation (SSF) offers a tangible alternative through the use of lignocellulosic wastes as support/substrate for the production of value-added products at feasible costs, showing significant advances in crucial fields, such as biofuels, enzymes, antibiotics, and organic acids production [7,8]. 

Fungi of the genus *Rhizopus* are generally saprophytes that colonize decaying or dead organic materials, such as leaves, husks, and soil. The most well-described member of the *Rhizopus* genus is *Rhizopus oryzae*, which is used to produce tempeh, a dish made from fermented soybeans. These microorganisms are instrumental in transforming agricultural and industrial residues into valuable bioproducts. *Rhizopus stolonifer*, commonly known as black bread mold, has extensive enzymatic capabilities, making it a promising candidate for biotechnological applications [9]. 

This study explores the potential of utilizing *Rhizopus stolonifer* to enrich CPH antioxidant properties and enhance its bioactive composition. By doing so, we seek to obtain a value-added product from this agricultural residue, contributing to its sustainable use and nutritional enhancement as a potential supplement to animal diets.

## 2. Materials and Methods

### 2.1. Cocoa Pod Husk Preparation

Cocoa fruits (*Theobroma cacao*, Forastero variety) were obtained in partnership with the Federal University of Pará (SisGen A4Ec257) from the Konagano cocoa farm (02°25′08″ S; 48°09′08″ W), located in the state of Pará, in the northern region of Brazil. Previously, the fruits were gently washed with distilled water to remove any debris after harvesting and shipment. CPHs were separated from the fruit pulp and dried in an oven with air circulation at 60 °C for 24 h. Subsequently, CPH was fragmented into smaller parts, ground in a knife mill, and separated into fractions using sieve sets ASTM No. 10 and No. 20, obtaining particles with sizes between 0.84–2.0 mm used in this study. 

### 2.2. Physicochemical Characterization

Total and reducing sugars were determined using the 2-hydroxy-3,5-dinitrobenzooic acid reaction (DNS) and a glucose standard curve (R^2^ = 0.9972) [10]. The Bradford method was used to determine the protein concentration using a bovine serum albumin (BSA) standard curve (R^2^ = 0.9911) [11]. The presence of ions was determined using Ion Chromatography 761 Compact IC (Metrohm AG, Herisau, Switzerland). Cations were analyzed with a Metrosep C3–250/4.0 column maintained at 40 °C, using a mobile phase of 3.5 mM HNO_3_ at a 0.9 mL min^−1^ flow rate. Anions were analyzed using a Metrosep A Supp 5–250/4.0 column at room temperature, with a mobile phase of 1 M sodium bicarbonate and 3.2 M sodium carbonate at a flow rate of 0.7 mL min^−1^. AquaLab CX-2 (Decagon Devices, Inc., Pullman, WA, USA) was used to measure the water activity (Aw). The pH was measured in a mixture obtained by homogenizing 1 g of the sample in 10 mL of distilled water using a pH meter mPA 210 (MS Tecnopon, Piracicaba, Brazil). Ash content was determined following the method proposed by the National Renewable Energy Laboratory [12], while humidity was analyzed using the methodology outlined by the Adolfo Lutz Institute [13].

### 2.3. Solid-State Fermentation

*Rhizopus stolonifer NRRL 28169* was acquired from the National Center for Agricultural Utilization Research (NRRL, Peoria, IL, USA) and maintained in the Department of Bioprocess Engineering and Biotechnology of the Federal University of Paraná. The strains were inoculated in medium Potato Dextrose Agar (PDA) for five days at 30 °C in Erlenmeyer flasks. Spores were harvested by submerging the culture in sterile distilled water containing 0.1% Tween 20. Spore concentrations were determined using a Neubauer chamber.

The physical conditions of SSF were based on the natural needs of the *Rhizopus* strains [14]. Erlenmeyer flasks containing 5 g of autoclaved CPH were inoculated with 5 mL of a spore suspension at a concentration of 10^7^ spores/mL. The initial pH of the CPH was 5.5 ± 0.1, with no adjustment. The moisture content was adjusted to 50% using distilled water, and the flasks were incubated and maintained at 30 °C. 

### 2.4. Extraction Methods

In this study, two different extraction methods were utilized to obtain different fractions of compounds. An aqueous ethanol extract (EAE) was obtained by mixing 5 g of sample from each flask in 50 mL of a 50% ethanol/DI water solution and agitated for one hour at 25 °C and alongside a method previously proposed by Conet et al. (2003) [15] and modified by Gonçalves et al. (2016) [16], where an acidified acetone (AAE) solution composed of acetone, water, and acetic acid (70:29.5:0.5 *v*/*v*) was used in three continuous steps on 5 g of sample in a total of 50 mL. After extraction, the suspension was filtered on the Whatman paper. All solvents from both extraction methods were evaporated in a vacuum kiln at 40 °C, and the samples were resuspended in ultrapure water for analyses.

### 2.5. Total Phenolic Content

Total phenolic content (TPC) was measured using the Folin-Ciocalteu method by Zheng and Wang (2001) [17], modified by Gouveia and Castilho (2011) [18]. The samples were dissolved in methanol to achieve a concentration (*w*/*v*) of 10 mg/mL. Aliquots of 50 μL were mixed with 1.25 mL of Folin-Ciocalteu reagent (diluted 1:10) and 1 mL of 7.5% sodium carbonate solution. After 30 min at room temperature, the decrease in absorbance was measured at 765 nm using a spectrophotometer. The results were quantified using a gallic acid standard curve (R^2^ = 0.9994) and expressed in gallic acid equivalent (GAE) per gram of sample (mg GAE g^−1^ sample).

### 2.6. Oxygen Radical Absorbance Capacity Assay and TEAC Calculation

The oxygen radical absorbance capacity (ORAC) was determined as described by Zulueta et al. (2009) [19]. The reaction was allowed to proceed for 30 min at 37 °C, and the absorbance was measured every minute to generate a decay curve of the absorbance value. The obtained values were then related to the Trolox standard curve, providing an ORAC result in μmol Trolox equivalence. The following equation was used for the calculations:

(1) $$ORAC\;\left(µmol\;TE\right)\frac{C\times k\times \left(AUCs-AUCb\right)}{\left(AUCt-AUCb\right)}$$

where *C* is the Trolox concentration (50 µM), *K* is the sample dilution factor, AUC is the area below the fluorescence decay curve of the sample (*AUCs*), blank (*AUCb*), and Trolox (*AUCt*) that is calculated by the following equation:

(2) $$AUC=\left(0.5+\frac{f5}{f0}+\frac{f10}{f0}\ldots +fn+\frac{5}{f0}\right)\times 5$$

where *f*0 is the initial fluorescence and *fn* is the fluorescence at time *n*. 

### 2.7. DPPH Radical Scavenging Activity and TEAC Calculation

The radical scavenging activity (RSA) of the extracts was measured according to the methodology presented by [20] with modifications. The reaction mixture consisted of 100 µL of sample (diluted to 1:20 *w*/*v*) added to 1.4 mL of DPPH methanol solution (100 µM). Absorbance was reset with distilled water and then read at 517 nm after 30 min of reaction using a UV-VIS spectrophotometer. The following equation was used to determine the scavenging activities of the samples:

$$RSA\;\left(\%\right)=\left(\frac{\left[{Abs}_{control}-{Abs}_{sample}\right]}{{Abs}_{control}}\right)\times 100$$

where *Abs_control_* is the absorbance of the sample eluent in DPPH solution. *Abs_sample_* is the absorbance of the sample in DPPH solution after 30 min of reaction.

A Trolox standard curve was prepared to estimate the sample’s Trolox equivalent antioxidant capacity (TEAC). Known concentrations of Trolox were subjected to the DPPH assay under identical conditions. The RSA values of Trolox were plotted against their concentrations to generate a standard curve (R^2^ = 0.9967).

### 2.8. Volatile Compounds Determination by GC-MS

The analysis of volatile compounds from the CPH and CPHF was performed using a headspace vial coupled to a solid phase microextraction (SPME) fiber (5% Carboxen [CARB]/95% Polydimethylsiloxane [PDMS], df 75 µm, partially cross-linked, Supelco, St. Louis, MI, USA). For each determination, 1 g of the sample was stored in a 20 mL HS vial. The flask was heated at 70 °C for 10 min without shaking, followed by 15 min of fiber exposure in the COMBI-PAL system to balance the volume in the vial. The compounds adsorbed by the fiber were desorbed into the gas chromatograph injection system gas phase (CGMS-gun TQ Series 8040 and 2010 Plus GC-MS Shimadzu, Tokyo, Japan) at 250 °C. The compounds were separated on a column containing 95% PDMS and 5% phenyl (30 m × 0.25 mm, 0.25 µm film thickness). The GC was equipped with an HP 5972 mass selective detector (Hewlett Packard Enterprise, Palo Alto, CA, USA). Helium was used as the carrier gas at a 1.0 mL/min rate. Mass spectra were obtained by electron impact at 70 eV. The compounds were identified by comparing the mass spectra those with in the library database (Nist’98 and Wiley7n) [21]. The same procedure was followed to perform the GC-MS analysis of the CPH and CPHF extracts (EAE and AAE). The extracts were completely dried and resuspended in ethanol P.A. HPLC grade. For each determination, 1 mL of the sample extract was stored in a 20 mL HS vial. The flask was heated at 50 °C for 10 min without shaking, followed by 15 min of fiber exposure in the COMBI-PAL system to balance the volume in the vial. The fiber exposure was reduced to 50 °C due to the ethanol boiling temperature (78 °C) to avoid fiber saturation.

### 2.9. Statistical Analysis

All measurements were performed in triplicate, and the data are presented as mean ± standard error of the mean. Statistical analysis was performed using two-way analysis of variance (ANOVA) to evaluate the effects of extraction method (EAE vs. AAE) and time on the DPPH values. Tukey’s Honestly Significant Difference (HSD) test was used for post-hoc comparisons to identify specific differences between the groups. The differences between the EAE and AAE methods at each time point were assessed using Student’s *t*-test. All statistical tests were conducted at a significance level of α = 0.05. Differences were considered statistically significant at *p* < 0.05. The analysis was performed using GraphPad Prism version 9.0.

## 3. Results

### 3.1. CPH Characterization

The physicochemical analysis of cocoa pod husk (Table 1) revealed a slightly acidic pH (6.18 ± 0.01), low water activity (0.283 ± 0.001), and minimal moisture content (3.4 ± 0.003%), indicating its stability and suitability for solid-state fermentation. CPH exhibited high nutritional potential, with notable levels of protein (18.30 ± 0.51 mg g^−1^), total sugars (17.347 ± 0.26 mg g^−1^), and reducing sugars (11.67 ± 0.15 mg g^−1^), which provide essential substrates for microbial metabolism. Its mineral composition, including sulfate (0.649 ± 0.029 mg g^−1^), phosphate (0.335 ± 0.010 mg g^−1^), and magnesium (0.440 ± 0.021 mg g^−1^) supports enzymatic activity and fungal growth. These results suggest that CPH is a nutritionally rich and stable substrate, making it an effective material for fermentation with *Rhizopus stolonifer* to enhance its functionality and nutrition.

### 3.2. SSF Kinetics

**Figure 1:**
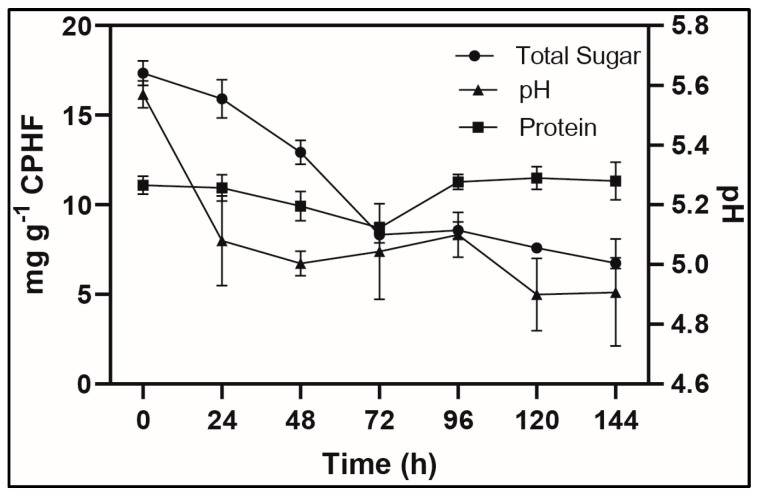
Kinetics of solid-state fermentation. Total sugar consumption, protein, and pH profile. Error bars represent the standard error values (*n* = 3).

CPH soluble protein content (18.30 ± 0.51 mg g^−1^) was reduced due to sterilization in autoclave, starting the SSF at 11.100 ± 0.417 with a content decrease to 8.707 mg at 72 h, but regaining soluble protein levels over 11 mg, reaching 11.327 ± 0.859 mg per g of CPHF at 144 h (Figure 1). Sugar content (total sugar: 17.347 ± 0.26 mg g^−1^, reducing sugar: 11.67 ± 0.15 mg g^−1^) provides essential nutrients for *R. stolonifer* metabolism. The slight variation in pH from 5.57 ± 0.05 (0 h) to 4.91 ± 0.18 (144 h) is ideal for *Rhizopus* spp. spore germination [22].

### 3.3. TPC

The TPC varied significantly across fermentation times and between the extraction methods (EAE and AAE). For dried CPH, the TPC was significantly higher with AAE (204.48 ± 1.27 mg GAE g^−1^) compared to EAE (125.00 ± 0.53 mg GAE g^−1^, *p* < 0.05), and both methods show how rich CPH is in its composition. At 0 h of SSF, the TPC decreased markedly for both methods due to sterilization in the autoclave, with EAE yielding 73.17 ± 0.84 mg GAE. g^−1^ and AAE yielding 82.39 ± 2.64 mg GAE. g^−1^, with no statistically significant difference between them. Throughout fermentation, the TPC fluctuated significantly. At 24 h, a peak in AAE was observed (134.18 ± 2.11 mg GAE. g^−1^) compared to that in EAE (87.69 ± 1.06 mg GAE. g^−1^, *p* < 0.05). This was followed by a substantial increase at 72 h for both methods, with AAE achieving 188.21 ± 0.53 mg GAE. g^−1^ and EAE achieving 114.70 ± 0.23 mg GAE. g^−1^ (*p* < 0.05). At 96 h, AAE maintained high TPC levels (122.84 ± 2.32 mg GAE. g^−1^) compared to EAE (71.08 ± 1.16 mg GAE. g^−1^, *p* < 0.05). The TPC plateaued at 120 h and declined slightly at 144 h for both methods, with AAE (92.69 ± 0.74 mg GAE. g^−1^) continuing to exhibit significantly higher values than EAE (75.90 ± 0.11 mg GAE. g^−1^) (Table 2). These results suggest that the AAE method consistently yielded higher TPC across all fermentation time points, with notable variations linked to time, likely due to the differential efficiency of phenolic compound release and degradation during extraction.

### 3.4. ORAC

The oxygen radical absorbance capacity (ORAC) assay results revealed a significant enhancement in antioxidant activity throughout the fermentation process. The dried cocoa pod husk (CPH) had an initial ORAC value of 47,723.04 ± 2796.90 µmol TE. g^−1^ sample, representing the SSF baseline. After 48 h, the value increased significantly to 50,312.11 ± 485.19 (*p* < 0.05). A steady upward trend was observed at 72, 96, and 120 h, showing progressive improvements, although not always statistically significant. At 144 h, the ORAC value peaked at 51,676.48 ± 347.99 µmol TE. g^−1^ sample, which was significantly higher than that of dried CPH (*p* < 0.05) (Table 3). These results suggest that SSF enhances the antioxidant capacity of the CPH.

### 3.5. TEAC

The antioxidant activity of cocoa pod husk (CPH) was assessed during solid-state fermentation at various time points using both EAE and AAE methods. Initial measurements at 0 h showed similar antioxidant activity, with 6.55 ± 0.05 µmol TE. g^−1^ sample for EAE and 6.72 ± 0.01 for AAE. Both extraction methods showed increased antioxidant activity as fermentation progressed, peaking at 96 h, with EAE measuring 7.09 ± 0.05 and AAE measuring 7.25 ± 0.04. A paired *t*-test analysis revealed that the differences in antioxidant activity between EAE and AAE were statistically significant at all time points (*p* < 0.05) (Table 4). These findings suggest that both extract fractions have an overall increase in antioxidant activity during SSF, confirming the findings observed in ORAC results. At the same time, AAE consistently reported higher values compared to EAE, with statistically significant differences observed throughout the process.

### 3.6. GC-MS

Volatile profile analysis of CPH and CPHF revealed several bioactive compounds with significant potential applications in the food, medical, and pharmaceutical industries. Notably, compounds such as α-methylbenzyl alcohol, 2-acetylpyrrole, aristolene, and rosefuran were exclusively identified in CPHF, along with methyl 3-hydroxybutyrate (M3HB), palmitic acid, and 10,12-tricosadiynoic acid. These bioactive compounds exhibit diverse biological activities, including antioxidant, anti-inflammatory, and therapeutic effects (Table 5).

## 4. Discussion

The physicochemical composition of CPH highlights its suitability for solid-state fermentation (SSF) by *Rhizopus stolonifer*. The slightly acidic pH (6.18 ± 0.01) and low water activity (Aw = 0.283 ± 0.001) create a favorable environment for fungal growth, requiring minimal adjustments [14]. The mineral profile, including sulfate (0.649 ± 0.029 mg g^−1^), phosphate (0.335 ± 0.010 mg g^−1^), and magnesium (0.440 ± 0.021 mg g^−1^), supports enzymatic activity and fungal growth [14]. These characteristics make CPH an ideal substrate for R. stolonifer to enhance its nutritional and functional properties through fermentation, demonstrating its potential for sustainable biotechnological applications (Table 1).

The TPC results indicated a higher phenolic content in the AAE fraction compared to theEAE fraction, with significant differences between the EAE and AAE methods across all time points (*p* < 0.05) (Table 2). AAE consistently yielded higher TPC values compared to EAE. After the CPH passed through sterilization at 120 °C in an autoclave for 20 min, all these physical factors, such as high pressure and temperature during thermal pretreatment, generated an approximate reduction of 59% in EAE and 40% in AAE from the initial TPC value [103,104]. The EAE fraction of dried CPH was 125.00 ± 0.53 mg GAE g^−1^, then fluctuated during SSF from 73.17 ± 0.84 at 0 h to its peak at 72 h, reaching 114.70 ± 0.23 mg GAE g^−1^. The AAE fraction showed a 204.48 ± 1.27 mg GAE g^−1^ of CPH before sterilization, starting SSF at 82.39 ± 2.64 mg GAE g^−1^, and it showed a similar pattern of fluctuation, with its peak at 72 h (188.21 ± 0.53 mg GAE g^−1^).

Previous work using the CPH showed lower values, reaching a range of 46–69 mg GAE g^−1^ [105]. Vriesmann et al. (2011) showed the presence of 46.0 ± 0.40 mg GAE g^−1^ of CPH from farms in Bahia, Brazil [106]. Yapo et al. (2013) registered 69.0 mg GAE/g CPH of fresh CPH from Côte d’Ivoire using four sequential solvents [107]. Even considering all the compositions of CPH from various areas and countries, the two methods presented in this work showed their efficiency in extracting phenolic compounds and support the hypothesis proposed in this study on how SSF using *Rhizopus stolonifer* can generate biomolecules with redox potential.

This study employed two complementary methods to assess antioxidant activity, ORAC and DPPH assays, to confirm the redox potential changes associated with biotransformation during SSF.

The ORAC assay revealed a significant increase in antioxidant activity due to SSF (Table 4). The initial ORAC value for CPH was (47.70 ± 0.28) × 10^3^ µmol TE g^−1^, which increased significantly to (51.70 ± 0.35) × 10^3^ µmol TE g^−1^ after 144 h (*p* < 0.05). Significant differences were observed between the CPH and all subsequent time points (*p* < 0.05). The ORAC values showed a gradual increase, with significant differences also noted between 48 h and both 120 h and 144 h (*p* < 0.05). The highest ORAC value was observed at 144 h, indicating a continuous increase in antioxidant activity during the extraction period.

The DPPH assay demonstrated significant differences between the EAE and AAE extraction methods across all time points (*p* < 0.05) (Table 3). For EAE, the DPPH values increased from 6.52 ± 0.03 µM TE g^−1^ at CPH to a peak of 7.09 ± 0.05 µM TE g^−1^ at 96 h before slightly decreasing. AAE showed a more consistent increase, starting at 6.60 ± 0.03 µM TE g^−1^ (CPH) and reaching a maximum of 7.25 ± 0.04 µM TE g^−1^ at 96 h after inoculation. Two-way ANOVA revealed significant main effects for both extraction method and time (*p* < 0.001), as well as a significant interaction between these factors (*p* < 0.01). AAE consistently yielded higher DPPH values compared to EAE across all time points.

The antioxidant activity observed in CPH is similar to that reported by Yapo et al. (2013), where approximately 85% of antioxidant activity was reported using fresh cocoa pod husk from the Ivory Coast [108]. The TEAC results obtained using the DPPH assay revealed significant differences between the EAE and AAE extraction methods. Consistently higher DPPH values for AAE at all time points indicate that this method may be more effective in extracting antioxidant compounds than EAE. This difference could be attributed to the varying selectivity of the two extraction methods for different antioxidant compounds in the sample [109].

For both extraction methods, the general trend of antioxidant activity peaked at 96 h of fermentation, followed by a slight decline. This pattern suggests an optimal fermentation duration during which antioxidant compounds can be degraded or reused. The increase in TEAC values confirmed the potential of *Rhizopus stolonifer* to enhance the antioxidant activity of CPH through SSF.

Analysis of the volatile profiles provided deeper insights into the antioxidant activity of CPH and CPHF and identified bioactive compounds of interest. The analysis revealed the presence of several bioactive compounds with potential application in food, medical, and pharmaceutical industries, such as α-methylbenzyl alcohol, 2-acetylpyrrole, aristolene, rosefuran, benzyl alcohol, 1-ethylpentyl acetate, 2-cyclopropylidene-1,7,7-trimethylbicyclo[2.2.1] heptane, β-hydroxyethyl phenyl ether, 2-hydrazino-2-imidazoline, methyl 3-hydroxybutyrate (M3HB), caprolactam, palmitic acid, and 10,12-tricosadiynoic acid which were all exclusively found in CPHF samples (Table 5). Beyond the antimicrobial, antioxidant, and anti-inflammatory activities of most of these compounds, SSF is justified by enhancing CPH with compounds like M3HB, which is known as an alternative energy source for cells with impaired metabolic function and has recently been reported to have therapeutic effects on Alzheimer’s disease and inhibition of apoptosis [73]. In addition, 10,12-tricosadiynoic acid, a specific Acyl-CoA Oxidase-1 inhibitor, enhances hepatic lipid metabolism and reduces oxidative stress by boosting mitochondrial fatty acid oxidation [110,111,112], and palmitic acid, which has shown anti-inflammatory potential and is an impactful animal diet supplement for the dairy industry [79,113].

Compounds known for their bioactive potential such as 2-methyl-n-hexacosane, ϒ-muurolene, isoledene, linalool, α-terpineol, 1-methoxy-3-(2-hydroxyethyl)nonane, heptanal, 1,2-benzenedicarboxylic acid, bis(2-methylpropyl) ester, were inevitably lost or metabolized by the *R. stolonifer* as most of these compounds present antifungal activities (Table 5). Furthermore, compounds like α-copaene, D-limonene, benzothiazole, β-ionone, and ethyl iso-allocholate were identified in CPH and CHPF, and are recognized for their anti-cancer effects, further highlighting the health-promoting potential of the fermented cocoa pod husk [66,99,114,115]. D-limonene is known for its antioxidant and antimicrobial properties [116].

Detection of complex compounds like cinnamic acid, 4-hydroxy-3-methoxy-(5-hydroxy-2-hydroxymethyl-6-[2-(4-hydroxy-3-methoxyphenyl)ethoxy]-4-(6-methyl-3,4,5-trihydroxytetrahydropyran-2-yloxy)tetrahydropyran-3-yl) ester, a derivative of ferulic acid (4-hydroxy-3-methoxycinnamic acid). This common plant-based phenolic acid is found in cell walls, bound to polysaccharides [66]. It has a sugar-like glycosylated structure with multiple hydroxyl and methoxy groups, which is typical of phenolic glycosides. This sugar-like structure attached to ferulic acid can influence its solubility, stability, and bioavailability, potentially increasing its antioxidant properties. These molecules can be transformed during fermentation, where microorganisms release bound ferulic acid and convert it into bioactive forms with enhanced antioxidant properties [117]. The presence of these compounds highlights the potential of CPH and CPHF as sources of bioactive compounds with therapeutic applications and as enriched nutritional supplements for animal feed.

## 5. Conclusions

This research demonstrates that solid-state fermentation with *Rhizopus stolonifer* can transform CPH, an agricultural waste product, into a valuable biomaterial with enhanced nutritional, antioxidant, and potential therapeutic properties, making CPHF a promising candidate for applications in food, animal feed, and bio-based industries. The physicochemical characterization of CPH revealed its suitability as a substrate for SSF, with fermentation improving protein recovery and significantly increasing antioxidant activity, as confirmed by ORAC and DPPH assays. These results highlight the redox potential of the biotransformation process, with the highest antioxidant activity observed at 144 h and 96 h.

GC-MS analysis identified a range of bioactive compounds, including methyl 3-hydroxybutyrate (M3HB), 10,12-Tricosadiynoic acid, and palmitic acid, which have applications in the food, pharmaceutical, and medical industries. Additionally, the preservation and transformation of phenolic compounds during SSF further enhanced the antioxidant profile of the fermented CPH.

This approach offers a sustainable solution for agricultural waste management and opens new avenues for developing functional food supplements, nutraceuticals, and potentially pharmaceutical ingredients. These findings underscore the potential of SSF to valorize agricultural byproducts, contributing to sustainable biotechnological innovations and the production of high-value biomaterials. Future studies will focus on pilot-scale experiments to evaluate production efficiency, product consistency, and potential commercial applications, while also incorporating kinetic modeling approaches to better understand and optimize the solid-state fermentation process.

## Figures and Tables

**Table 1:** Physicochemical composition of CPH.

Analysis	Value in CPH
pH	6.18 ± 0.01
A_w_	0.283 ± 0.001
Ashes Content (%)	8.2 ± 0.04
Humidity (%)	3.4 ± 0.003
	Value (mg g^−1^)
Total sugar	17.347 ± 0.26
Reducing sugar	11.67 ± 0.15
Protein	18.30 ± 0.51
F^−^	0.114 ± 0.009
Cl^−^	0.318 ± 0.015
Br^−^	0.198 ± 0.008
NO_2_^−^	0.359 ± 0.005
SO_4_^−2^	0.649 ± 0.029
PO_4_^−3^	0.335 ± 0.010
Na^+^	0.022 ± 0.003
NH_4_^+^	0.080 ± 0.010
K^+^	0.200 ± 0.006
Mg^+^	0.440 ± 0.021
Ca^+^	0.032 ± 0.009

**Table 2:** Total phenolic content (TPC) of cocoa pod husk for AAE and EAE methods during fermentation (values with an asterisk (*) indicate a statistically significant difference between AAE and EAE, *p* < 0.05).

Fermentation Time (h)	TPC (mg GAE. g^−1^ Sample)	
	EAE	AAE
Dried CPH	125.00 ± 0.53 ^e^*	204.48 ± 1.27 ^g^
0 h	73.17 ± 0.84 ^a^*	82.39 ± 2.64 ^a^
24 h	87.69 ± 1.06 ^c^*	134.18 ± 2.11 ^e^
48 h	85.26 ± 2.10 ^c^*	115.52 ± 0.63 ^c^
72 h	114.70 ± 0.23 ^d^*	188.21 ± 0.53 ^f^
96 h	71.08 ± 1.16 ^a^*	122.84 ± 2.32 ^d^
120 h	82.09 ± 1.48 ^bc^*	137.61 ± 1.16 ^e^
144 h	75.90 ± 0.11 ^ab^*	92.69 ± 0.74 ^b^

Note: Values are presented as mean ± standard error. Different superscript letters (a–g) within each column indicate statistically significant differences (*p* < 0.05) based on two-way ANOVA, followed by Tukey’s HSD test. Asterisks (*) indicate significant differences between EAE and AAE methods for each time point (*p* < 0.05).

**Table 3:** Oxygen radical absorbance capacity (ORAC, Trolox equivalent—TE) of EAE from cocoa pod husk during fermentation (48 to 144 h).

Fermentation Time (h)	ORAC (µmol TE. g^−1^ Sample)
Dried CPH	47,723.04 ± 2796.90 ^a^
48 h	50,312.11 ± 485.19 ^b^
72 h	50,391.40 ± 4501.17 ^bc^
96 h	50,535.57 ± 800.70 ^bc^
120 h	51,645.00 ± 76.93 ^cd^
144 h	51,676.48 ± 347.99 ^d^

Note: Values are presented as mean ± standard error. Different superscript letters (a, b, c, d) indicate statistically significant differences (*p* < 0.05) based on one-way ANOVA, followed by Tukey’s HSD test.

**Table 4:** Trolox equivalent antioxidant capacity (TEAC) measured by DPPH Assay in µM TE per gram of cocoa pod husk for AAE and EAE methods during fermentation.

Fermentation Time (h)	TEAC (µmol TE. g^−1^ Sample)	
	EAE	AAE
Dried CPH	6.52 ± 0.03 ^a^*	6.60 ± 0.03 ^a^
0	6.55 ± 0.05 ^a^*	6.72 ± 0.01 ^b^
24	6.56 ± 0.05 ^a^*	6.95 ± 0.02 ^c^
48	6.64 ± 0.08 ^ab^*	7.10 ± 0.02 ^d^
72	6.73 ± 0.09 ^bc^*	7.15 ± 0.01 ^de^
96	7.09 ± 0.05 ^e^*	7.25 ± 0.04 ^f^
120	6.98 ± 0.01 ^de^*	7.17 ± 0.05 ^ef^
144	6.93 ± 0.09 ^cd^*	7.09 ± 0.03 ^d^

Note: Values are presented as mean ± standard error. Different superscript letters (a–f) within each column indicate statistically significant differences (*p* < 0.05) based on a two-way ANOVA followed by Tukey’s HSD test. Asterisks (*) indicate significant differences between EAE and AAE methods for each time point (*p* < 0.05).

**Table 5:** Volatile compounds identified in non-extracted CPH and CPHF after 96 h of fermentation, acidified acetone extract (AAE), and ethanol aqueous extract (EAE) from cocoa pod husk and cocoa pod husk After 48, 96, and 144 hours of fermentation by gas chromatography coupled to mass spectrometry (GC-MS).

			Solid-State		EAE				AAE				Bioactivity	Reference
Alcohol	Formula	Mol Wt	CPH	CPHF	CPH	48 h	96 h	144 h	CPH	48 h	96 h	144 h		
2,3-Butanediol	C_4_H_10_O_2_	90	-	+	-	-	-	-	-	-	-	-	Fuel additive	[23]
1-Hexanol	C_6_H_14_O	102	+	-	-	-	-	-	-	-	-	-	Aroma	[24]
(2S,4S)-(+)-Pentanediol	C_5_H_12_O_2_	104	+	+	-	-	-	-	-	-	-	-	N/A	
1,2-Pentanediol	C_5_H_12_O_2_	104	-	+	-	-	-	-	-	-	-	-	N/A	
1-Methoxy-2-butanol	C_5_H_12_O_2_	104	+	+	-	-	-	-	-	-	-	-	N/A	
2-Ethoxy-1-propanol	C_5_H_12_O_2_	104	-	-	-	-	-	-	+	-	-	-	N/A	
Benzyl alcohol	C_7_H_8_O	108	-	+	-	-	-	-	-	-	-	-	Antimicrobial	[25]
3,4-Dimethylpent-2-en-1-ol	C_7_H_14_O	114	-	+	-	-	-	-	-	-	-	-	N/A	
5-Methyl-2-hexanol	C_7_H_16_O	116	-	+	-	-	-	-	-	-	-	-	Aroma	[26]
2-Methyl-3-hexanol	C_7_H_16_O	116	-	+	-	-	-	-	-	-	-	-	N/A	
2-Ethyl-3-pentanol	C_7_H_16_O	116	+	-	-	-	-	-	-	-	-	-	N/A	
Hexylene glycol	C_6_H_14_O_2_	118	-	-	+	-	+	+	-	-	-	+	Antibacterial & Antifungal	[27]
Phenylethyl Alcohol	C_8_H_10_O	122	+	+	-	-	-	-	-	-	-	-	Antifungal & Aroma	[28]
α-Methylbenzyl alcohol	C_8_H_10_O	122	-	+	-	-	-	-	-	-	-	-	Aroma	[29]
1-Octen-3-ol	C_8_H_16_O	128	+	-	-	-	-	-	-	-	-	-	Food additive	[30]
2,6-Dimethylcyclohexanol	C_8_H_16_O	128	+	-	-	-	-	-	-	-	-	-	Anesthetic	[31]
2-Ethyl-1-hexanol	C_8_H_18_O	130	+	-	-	-	-	-	-	-	-	-	Aroma	[32]
1-Nonanol	C_9_H_20_O	144	+	-	-	-	-	-	-	-	-	-	Antifungal & Aroma	[33]
1-Phenoxy-2-propanol	C_9_H_12_O_2_	152	-	-	-	-	+	-	-	-	-	-	Anesthetic	[34]
3-Phenoxy-1-propanol	C_9_H_12_O_2_	152	-	-	-	-	+	-	-	-	-	-	Aroma	[35]
Linalool	C_10_H_18_O	154	+	-	-	-	-	-	-	-	-	-	Anti-inflammatory & Anticonvulsant	[36]
α-Terpineol	C_10_H_18_O	154	+	-	-	-	-	-	-	-	-	-	Antifungal	[37]
[5-Hydroxymethyl)-1,3-dioxolan-4-yl]methanol	C_5_H_10_O_4_	134	-	-	-	-	-	+	+	-	-	-	N/A	
Cis-Linalool oxide	C_10_H_18_O_2_	170	+	+	-	-	-	-	-	-	-	-	Aroma	[38]
Linalool oxide pyranoid	C_10_H_18_O_2_	170	+	+	-	-	-	-	-	-	-	-	Aroma	[39]
Trans-furanoid linalool oxide	C_10_H_18_O	170	+	-	-	-	-	-	-	-	-	-	Aroma	[40]
11-Methyldodecanol	C_13_H_28_O	200	+	-	-	-	-	-	-	-	-	-	N/A	
1-Tridecanol	C_13_H_28_O	200	+	-	-	-	-	-	-	-	-	-	N/A	
1-Methoxy-3-(2-hydroxyethyl)nonane	C_12_H_26_O_2_	202	-	-	+	-	-	-	-	-	-	-	Antioxidant & Antifungal	[41,42]
2,2-Dimethyl-6-methylene-1-[3,5-dihydroxy-1-pentenyl]cyclohexan-1-perhydrol	C_14_H_24_O_4_	256	-	-	-	-	-	-	-	-	+	-	Antioxidant, Anti-inflammatory, Antidiabetic, Antitumor, etc	[43,44]
Aldehyde														
(E,E)-2,4-Heptadienal	C_7_H_10_O	110	+	-	-	-	-	-	-	-	-	-	Antifungal & Aroma	[45]
Heptanal	C_7_H_14_O	114	+	-	-	-	-	-	-	-	-	-	Antifungal	[46]
Methoxycitronellal	C_11_H_22_O_2_	186	-	+	-	-	-	-	-	-	-	-	Aroma	[47]
Nonanal	C_9_H_18_O	142	+	+	-	-	-	-	-	-	-	-	Antifungal	[48]
Octanal	C_8_H_16_O	128	+	+	-	-	-	-	-	-	-	-	Antifungal	[49]
(E)-2-hexenal	C_6_H_10_O	98	+	-	-	-	-	-	-	-	-	-	Antifungal	[50]
(E)-2-Octenal	C_8_H_14_O	126	+	-	-	-	-	-	-	-	-	-	Antifungal	[51]
Phenylacetaldehyde	C_8_H_8_O	120	+	+	-	-	-	-	-	-	-	-	Aroma	[52]
β-Cyclocitral	C_10_H_16_O	152	+	-	-	-	-	-	-	-	-	-	Antibacterial	[53]
Ketone														
2-Heptanone	C_7_H_14_O	114	+	-	-	-	-	-	-	-	-	-	Neuromodulation	[54]
Geranylacetone	C_13_H_22_O	196	+	-	-	+	-	-	-	-	-	-	Aroma	[55]
Acetophenone	C_8_H_8_O	87	+	-	-	-	-	-	-	-	-	-	Aroma & Food additive	[56]
2-Dodecanone	C_12_H_24_O	184	+	-	-	-	-	-	-	-	-	-	Insecticidal & Repellent	[57]
1,3-Diacetylbenzene	C_10_H_10_O_2_	162	-	-	-	-	+	+	+	-	-	+	N/A	
1,4-Diacetylbenzene	C_10_H_10_O_2_	162	-	-	+	-	+	+	+	-	+	+	N/A	
2-Acetylpyrrole	C_6_H_7_NO	109	-	+	-	-	-	-	-	-	-	-	Antioxidant & Hepatoprotective	[58]
Sulcatone	C_8_H_14_O	126	+	-	-	-	-	-	-	-	-	-	Antimicrobial	[59]
trans-3-Octen-2-one	C_8_H_14_O	126	+	-	-	-	-	-	-	-	-	-	Aroma	[60]
β-Ionone	C_13_H_20_O	192	+	-	-	-	-	-	-	-	+	-	Aroma, Antimicrobial & Insecticidal	[61]
Ester														
1,2-Benzenedicarboxylic acid, bis(2-methylpropyl) ester	C_16_H_22_O_4_	278	-	-	-	-	-	-	+	-	-	-	Antioxidant & Antimicrobial	[62]
1,2-Dimethylpropyl acetate	C_7_H_14_O_2_	130	+	-	-	-	-	-	-	-	-	-	N/A	
1-Ethylpentyl acetate	C_9_H_18_O_2_	158	-	+	-	-	-	-	-	-	-	-	Antimicrobial	[63]
2-(Heptyloxycarbonyl)benzoic acid	C_15_H_20_O_4_	264	-	-	-	-	+	+	+	+	-	-	Antiobesity & Anti-hyperlipidemic	[64]
Triethylene glycol dimethacrylate	C_14_H_22_O_6_	286	-	-	-	-	-	-	+	+	-	-	Cytotoxic (mammalian cells)	[65]
Cinnamic acid, 4-hydroxy-3-methoxy-,(5-hydroxy-2-hydroxymethyl-6-[2-(4-hydroxy-3-methoxyphenyl)ethoxy]-4-(6-methyl-3,4,5-trihydroxytetrahydropyran-2-yloxy)tetrahydropyran-3-yl) ester	C_31_H_40_O_15_	652	-	-	+	+	+	+	+	+	-	+	Antioxidant & Antiviral	[66]
Dimethyl phthalate	C_10_H_10_O_4_	194	-	-	+	-	+	+	-	-	-	+	Environmental Contaminator	[67]
Dodecanoic acid, 2,3-bis(acetyloxy)propyl ester	C_19_H_34_O_6_	358	-	-	+	-	-	-	-	+	-	-	Antiviral	[68]
Ethyl iso-allocholate	C_26_H_44_O_5_	452	+	+	+	+	+	+	+	+	+	+	Antiangiogenic	[69]
Fumaric acid, 2-isopropyl phenyl dodec-2-en-1-yl ester	C_25_H_36_O_4_	400	-	-	-	-	-	-	-	-	+	-	Antioxidant, Immunomodulating, and Anti-inflammatory	[70]
Methyl salicylate	C_8_H_8_O_3_	152	+	+	-	-	-	-	-	-	-	-	Anti-inflammatory & Analgesic agent	[71]
Octacosanoic acid, methyl ester	C_29_H_58_O_2_	438	-	-	-	-	-	-	-	+	-	-	N/A	
Oxalic acid, bis(6-ethyloct-3-yl) ester	C_22_H_42_O_4_	370	+	-	-	-	-	-	-	-	-	-	N/A	
Methoxy-Phenyl-Oxime	C_8_H_9_NO_2_	151	+	+	-	+	+	+	+	+	+	+	Antibacterial	[72]
Methyl 3-hydroxybutyrate	C_5_H_10_O_3_	118	-	+	-	-	-	-	-	-	-	-	Therapeutic effect on Alzheimer’s disease & Inhibition of apoptosis	[73]
ϒ-Butyrolactone	C_4_H_6_O_2_	86	+	-	-	-	-	-	-	-	-	-	Regulate Antibiotic production in Streptomyces	[74]
Pantolactone	C_6_H_10_O_3_	130	+	-	-	-	-	-	-	-	-	-	Food additive	[75]
Mevalonolactone	C_6_H_10_O_3_	130	-	+	-	-	-	-	-	-	-	-	Mevalonate pathway precursor (3- Hydroxy-3-methylglutaryl Coenzyme A Reductase inhibitor)	[76]
3,3,5-trimethylcyclohexyl methacrylate	C_13_H_22_O_2_	210	-	-	+	+	-	-	-	-	-	-	N/A	
Organic acid														
10,12-Tricosadiynoic acid	C_23_H_38_O_2_	346	-	+	-	-	-	-	-	-	-	-	ACOX1-specific inhibitor	[77]
Isovaleric acid	C_5_H_10_O_2_	102	+	-	-	-	-	-	-	-	-	-	Colonic Smooth Muscle Relaxation	[78]
Palmitic acid	C_16_H_32_O_2_	256	-	-	-	-	-	-	-	+	-	-	Diet Supplement for animal	[79]
Tetradecanoic acid	C_14_H_28_O_2_	228	-	-	-	-	-	-	-	+	-	-	Larvicidal & mosquito repellent	[80]
Terpenes														
Aristolene	C_15_H_24_	204	-	+	-	-	-	-	-	-	-	-	Biopesticidal, Anti-inflammatory, Antidiabetic, Anti-urolithic, and Tyrosinase inhibitory potentials	[81,82]
1-Isopropyl-4,7-dimethyl-1,2,3,5,6,8a-hexahydronaphthalene	C_15_H_24_	204	+	+	-	-	-	-	-	-	-	-	Antioxidant & Antibacterial	[83]
Isoledene	C_15_H_24_	204	+	-	-	-	-	-	-	-	-	-	Antiviral & Anti-leishmania	[84]
Valerena-4,7(11)-diene	C_15_H_24_	204	+	-	-	-	-	-	-	-	-	-	Sedative effect	[85]
α-Copaene	C_15_H_24_	204	+	+	-	-	-	-	-	-	-	-	Antimicrobial	[86]
ϒ-Cadinene	C_15_H_24_	204	+	-	-	-	-	-	-	-	-	-	Antioxidant & Anti-inflammatory	[87]
ϒ-Muurolene	C_15_H_24_	204	+	-	-	-	-	-	-	-	-	-	Antioxidant & Anti-inflammatory	[88]
D-Limonene	C_10_H_16_	136	+	+	-	-	-	-	-	-	-	-	Anti-inflammatory & Antibiofilm	[89]
Rosefuran	C_10_H_14_O	150	-	+	-	-	-	-	-	-	-	-	Antioxidant	[90]
Hydrocarbon														
1,2,4-Trimethylcyclopentane	C_8_H_16_	112	-	+	-	-	-	-	-	-	-	-	N/A	
13-Phenylpentacosane	C_31_H_56_	428	-	+	-	-	-	-	-	-	-	-	N/A	
1-Heptadecene	C_17_H_34_	238	+	-	-	-	-	-	-	-	-	-	N/A	
2,6,10,15-Tetramethylheptadecane	C_21_H_44_	296	+	-	-	-	-	-	-	-	-	-	Multiple therapeutic potentialities	[91]
2-Cyclopropylidene-1,7,7-trimethylbicyclo [2.2.1]heptane	C_13_H_20_	176	-	+	-	-	-	-	-	-	-	-	Antimicrobial	[92]
2-Ethylhexene	C_8_H_16_	112	-	+	-	-	-	-	-	-	-	-	N/A	
2-Methyl-n-hexacosane	C_27_H_56_	380	+	-	-	-	-	-	-	-	-	-	Anti-Carcinoma Cell Growth	[93]
Eicosane	C_20_H_42_	282	+	-	-	-	-	-	-	-	-	-	Antifungal, Antioxidant, and Anti-inflammatory	[94,95]
Heneicosane	C_21_H_44_	296	+	-	-	-	-	-	-	-	-	-	Antimicrobial	[96]
Nitrile														
Octanenitrile	C_8_H_15_N	125	+	-	-	-	-	-	-	-	-	-	Aroma	[97]
Amine														
Putrescine	C_4_H_12_N_2_	88	+	-	-	-	-	-	-	-	-	-	N/A	
2-Hydrazino-2-imidazoline	C_3_H_8_N_4_	100	-	-	-	-	-	-	-	+	-	-	Antioxidant	[98]
Heterocyclic														
Benzothiazole	C_7_H_5_NS	135	-	-	+	-	+	+	+	-	+	+	Antimicrobial, Anticonvulsant, Neuroprotective, Anti-inflammatory, and Antitumor	[99]
Caffeine	C_8_H_10_N_4_O_2_	194	-	-	-	-	-	-	+	-	-	-	Alleviates fatigue and drowsiness	[100]
Amide														
Caprolactam	C_6_H_11_NO	113	-	-	-	-	-	+	-	-	-	-	Caspase-1 inhibitor	[101]
Ether														
β-Hydroxyethyl phenyl ether	C_8_H_10_O_2_	138	-	-	-	-	+	-	-	-	+	-	Antibacterial & Antifungal	[102]

Note: (+) Presence or (-) Absence. N/A—Bioactivity not found in the literature.

## Data Availability

The original contributions presented in this study are included in the article. Further inquiries can be directed to the corresponding author.
